# Piecewise power laws in individual learning curves

**DOI:** 10.3758/s13423-015-0811-x

**Published:** 2015-02-25

**Authors:** Yoni Donner, Joseph L. Hardy

**Affiliations:** 1 Department of Computer Science, Stanford University, Stanford, California 94305 USA; 2 Department of Research and Development, Lumos Labs, San Francisco, CA 94108 USA

**Keywords:** Individual learning curves, Power law, Strategy shifts, Computational models, Skill acquisition, Skill learning, Cognitive training

## Abstract

The notion that human learning follows a smooth power law (PL) of diminishing gains is well-established in psychology. This characteristic is observed when multiple curves are averaged, potentially masking more complex dynamics underpinning the curves of individual learners. Here, we analyzed 25,280 individual learning curves, each comprising 500 measurements of cognitive performance taken from four cognitive tasks. A piecewise PL (PPL) model explained the individual learning curves significantly better than a single PL, controlling for model complexity. The PPL model allows for multiple PLs connected at different points in the learning process. We also explored the transition dynamics between PL curve component pieces. Performance in later pieces typically surpassed that in earlier pieces, after a brief drop in performance at the transition point. The transition rate was negatively associated with age, even after controlling for overall performance. Our results suggest at least two processes at work in individual learning curves: locally, a gradual, smooth improvement, with diminishing gains within a specific strategy, which is modeled well as a PL; and globally, a discrete sequence of strategy shifts, in which each strategy is better in the long term than the ones preceding it. The piecewise extension of the classic PL of practice has implications for both individual skill acquisition and theories of learning.

The power law (PL) of practice (Newell & Rosenbloom, 1981) quantitatively describes a smooth time course of diminishing gains (Wright, 1936) in learning. It is supported by observations that learning curve averages are well-fit by smooth PLs, and it appears in well-known cognitive architectures (Anderson et al., 2004). However, rather than representing the underlying neurocognitive process, the PL characteristic of learning curves may be an artifact of averaging across individual curves (Estes, 1956; Gallistel, Fairhurst, & Balsam, 2004; Haider & Frensch, 2002; Heathcote, Brown, & Mewhort, 2000; Myung, Kim, & Pitt, 2000); using a single PL (PL1) to predict individual performance may obscure more complex learning dynamics (Delaney, Reder, Staszewski, & Ritter, 1998; Rickard, 1999).

Punctuated dynamics of individual curves have been observed in formal studies of learning since the 19th century (Bryan & Harter, 1899). Whether the formation of associations is incremental or binary is still debated (Rock, 1957; Roediger, 2012). Individual learning curves in several different animal conditioning experiments have included an abrupt, step-like increase (Gallistel et al., 2004). In humans, punctuated dynamics have been observed in concept learning (Bruner, Goodnow, & Austin, 1986), visual task learning (Karni & Sagi, 1993), and “aha!” moments (Kounios & Beeman, 2009). Such observations are consistent with the intuition that skill acquisition involves both plateaus and bursts of rapid improvement (Gregory, 1968).

Despite observations of punctuated learning dynamics across multiple domains, smooth, unitary PL models are still the norm in quantitative analysis (Anderson, 2000), with a few important exceptions. Gallistel et al. (2004) have argued that such smooth PLs (or Weibull functions) are an artifact of averaging. Using multiple data sets of animal conditioning data, they demonstrated abrupt changes in the slopes of the cumulative number of learned conditioned responses. They provided strong evidence that individual-learning curves are not smooth PLs and suggested an algorithm for change-point detection, but they did not provide a full quantitative model for entire individual-learning curves. Furthermore, their results may not extend beyond animal conditioning. Heathcote et al. (2000) also argued that smooth PL curves are an artifact of averaging and proposed alternative models for individual curves. On the basis of their work (Heathcote et al., 2000), we included three-parameter exponential functions in our analysis. Delaney et al. (1998) described a model in which different PLs correspond to different strategies within the learning curve, and Rickard proposed component power laws (CMPL) supported by both theoretical arguments and empirical data (Rickard, 1999, 2004). Like both strategy-specific PLs and CMPL, the piecewise PLs (PPLs) presented here combine multiple PLs in individual learning curves. Without assuming known strategies or fixed PL components, we focused on identifying the PL transitions and properties of strategy shifts.

Distinguishing true discontinuities from noisy but fundamentally smooth learning curves requires large data sets with learning curves from many individuals. Moreover, the individual curves should be long enough to capture details of the learning dynamics and should include potentially infrequent discontinuities. These requirements are even more critical for characterizing the properties of discontinuities. Such data have been lacking from studies of human learning curves.

Here, we analyzed a new data set from human learning in cognitive tasks (Hardy, Farzin, & Scanlon, 2013) that is much larger than the data sets previously used in learning curve analyses. Using new procedures for fitting and model selection, we demonstrate that shifts between PLs occur in individual learning curves, and we describe a global, two-process view of improvement on a task.

## Method

### Data

Learning curve data were collected from versions of four well-known cognitive tasks included in the online cognitive-training program Lumosity (www.lumosity.com; Lumos Labs, San Francisco, CA). Cognitive performance data collected online can be as valid as data collected in laboratory settings (Germine et al., 2012). As of September 16, 2014, the Lumosity database contained data from 2,130,428,156 exercise completions from 65,357,911 registered users worldwide, engaging with over 50 exercises. The tasks included one-back speed-of-processing (1B, *Speed Match*), two-back memory updating (2B, *Memory Match*), Eriksen flanker (EF, *Lost in Migration*), and verbal fluency (VF, *Word Bubbles*) tasks. Data from individuals with at least 500 completions for a task were included (*n* = 25,280 learning curves, 22,460 unique participants). A total of 20,527 (91.4 %) participants had a learning curve included for only one task, and 1,232 (5.5 %), 515 (2.3 %), and 186 (0.8 %), respectively, had curves for two, three, and all four tasks.

The participants were able, but not required, to provide demographic information. In all, 13,933 participants provided their gender, education level, country, and age between 18 and 80. Most were female (10,254, 73.6 %). The average age was a bit higher for females (females: 52.2 ± 12.36 years, males: 47.32 ± 13.8 years, overall: 50.92 ± 12.94 years); 57.8 % of the participants specified academic degrees (31.63 % bachelors, 21.47 % masters, 4.74 % PhD), and over 65 % specified their country as the USA.

### Tasks

Speed Match is a one-back speed-of-processing task in which the user indicates whether the current symbol matches the previous one. Several different sets of symbols are used, including simple colored circles, squares, and triangles, as well as more complex target symbols and kanji characters. Memory Match is a two-back visual working memory task (e.g., Buschkuehl & Jaeggi, 2010) in which users indicate whether the current symbol matches the one presented two symbols previously. Lost in Migration utilizes an Eriksen (1995) flanker response inhibition task: In each trial, five birds are presented in a pattern resembling a flock in flight. The user’s task is to indicate the direction of the middle bird in a four-alternative forced choice response. In congruent trials, the direction of the middle bird is the same as that of the other birds, and in incongruent trials it is different. The scores for the 1B, 2B, and EF tasks are based on the numbers of correct answers provided in 45 s. Word Bubbles is a verbal fluency task (see, e.g., Ruff, Light, Parker, & Levin, 1997) in which the user generates as many words as possible from a given three-letter word stem. Each exercise session comprises three word stems to be completed within 60 s. These tasks are described more thoroughly elsewhere (Kesler et al., 2013; Mayas, Parmentier, Andrés, & Ballesteros, 2014; Sternberg et al., 2013).

### Data processing

Since using raw scores directly could make the results distribution-specific, scores were first normalized by rank-based inverse normal transformation (Van der Waerden, 1952). The transformation was applied separately for each task and across all participants and positions on the learning curve. The transformed data distribution for each task was normal with zero mean and unit variance.

Outliers were identified using a probabilistic model that allowed for the detection of infrequent (prior probability .05) low-score outliers, including multiple consecutive ones, in an otherwise continuous learning curve that was modeled as a random walk with variance .25. The outlier model was Gaussian with mean −1.5 and unit variance. Since the posterior outlier probability of a point depends on the previous nonoutlier point, a dynamic programming algorithm similar to “forward–backward” (Rabiner & Juang, 1986) was required in order to compute the posterior outlier probabilities. Points with posterior outlier probabilities above .25 were removed. These parameters were chosen by manual examination of the data.

This method removes points lacking support on either side for being part of the continuous curve. It removes discontinuities, but cannot add false ones. Less than 2 % of the scores were removed. The first 500 scores in curves comprising at least 500 exercises were used in the analysis (*n* = 25,280).

### Power law fitting

A four-parameter PL was used (Newell & Rosenbloom, 1981): *f*(*t*) = *u* – *a*(*t* + *d*)^*c*^, with asymptote *u*, slope *a*, power *c*, and delay *d*. The *c* and *d* were specified as exponents (*c* = −*e* ^*c'*^, *d* = *e* ^*d'*^) to constrain *c* < 0, *d* > 0, but *a* was not constrained to be positive, allowing for decreasing curves. Fitting was done using Newton’s method (Boyd, 2004) in order to minimize the squared error ||*y*(*t*) – *f*(*t*)||^2^.

Three-parameter PLs with no *d* parameter, *f*(*t*) = *u* – *at* ^*c*^, and three-parameter exponential functions (Heathcote et al., 2000), *f*(*t*) = *u* – *ae* ^*ct*^, were fit as well. Brent’s method for optimizing over a single variable (Brent, 1972) was used to fit the power *c*, with least-squares to compute the squared error for each value of *c*.

### Piecewise power law definition and fitting

In the PPL model, multiple PL pieces are combined to fit a single learning curve. The PPLs define *k* – 1 transition points for *k* pieces covering the entire curve, where each piece is fit by a PL. To fit the PPLs, individual PLs were fit to all windows of 50 or more consecutive scores within the learning curve. We did not fit shorter windows, in order to improve parameter robustness and avoid overfitting small segments. This is conservative, since fitting shorter windows could only increase the number of pieces. To speed up running times, warm start (initialization with a previous solution) was used to extend the fits by one position. After computing squared errors for each window, a dynamic programming algorithm (Bellman & Rand Corp., 1957) was used to compute, for each number of pieces, the optimal transition points to minimize the total error on the entire curve. This dynamic programming procedure is an efficient way to find optimal transition points between the PL pieces, instead of brute-force search. Since PPL fitting is an essential part of our work, we provide Python code for the fitting procedure in the Appendix.

### Choice of model selection procedure

Fitting PPLs involves comparing models of different complexities. Our analysis requires accurate and conservative model selection. We use the machine-learning terms “underfitting” and “overfitting” to indicate choosing too few or too many pieces, respectively. Unlike overfitting, underfitting does not undermine our conclusion that real-life individual learning curves comprise multiple PL pieces, but it can mask the true PL transitions. We use “conservative” to describe analysis methods that do not increase the likelihood of producing results supporting our PPL hypothesis.

Our fitting algorithm computes the best sequence of PL pieces for every model size (number of pieces). To prevent overfitting, three common criteria (the Akaike information criterion [AIC], small-sample-corrected AIC [AICc], and Bayesian information criterion [BIC]) for controlling model complexity (Burnham, 2004) were evaluated in a simulation study (the simulation procedure is described below). A PPL with *m* pieces uses 5 *m* – 1 parameters (i.e., 4 *m* PL parameters and *m* – 1 transition points). We used several evaluation metrics, using either the best-scoring model or Akaike weights, which define a probability distribution over models based on the penalized likelihood (Burnham, 2004).

The following notation is used to describe the metric for evaluating our model selection: The curves are indexed as 1 ≤ *i* ≤ *n*. Akaike weights *w*[*i*, *j*] define the probability, derived from the penalized likelihood, of curve *i* having *j* pieces. The most likely number of pieces corresponds to the highest Akaike weight: *m*[*i*] = argmax_*j*_ *w*[*i*, *j*]. The number of pieces used to simulate the *i*th curve is *k*[*i*]*.* Our metrics average, over the simulation data, the curve-specific metrics described in Table 1, and lower numbers indicate higher accuracy.

**Table 1:** Six metrics used to evaluate model selection methods on simulated data for which the true number of pieces *k*[*i*] is known

Metric	Formula	Description
LL	–log(*w*[*i*,*k*[*i*]])	Negative log-likelihood of the data from the Akaike weights
E1	(*k*[*i*] – *m*[*i*])^2^	Squared error for the best model choice
E2	(*k*[*i*] – ∑*jw*[*i*, *j*])^2^	Squared error for the expectation of model choice
ER	1 if *k*[*i*] ≠ *m*[*i*], else 0	Error fraction
FP	1 if *k*[*i*] = 1 and *m*[*i*] > 1	False positives (multiple-piece instead of single-piece)
FN	1 if *k*[*i*] > 1 and *m*[*i*] = 1	False negatives (single-piece instead of multiple-piece)

In addition to evaluating model selection methods based on choosing the right number of pieces, we evaluated the predictive accuracy on future values, using accumulative prediction error (APE), a data-driven model selection criterion for time series (Wagenmakers, Grünwald, & Steyvers, 2006). For computational reasons, APE was evaluated on a random subset of 1,000 learning curves chosen uniformly at random from the entire data.

### Simulated data

Simulated PPL data were used to evaluate the model selection procedures described above. A total of 1,000 curves were generated for each of one to four pieces (4,000 total simulated curves). The piece lengths were sampled from an exponential distribution (*λ* = 0.005) using rejection sampling to condition on the desired number of pieces. Due to this conditioning, the rate *λ* was not critical (e.g., with two pieces, the average piece length was 250, regardless of the exponential rate parameter). PL parameters for each piece were estimated on the real learning curve data.

The pieces were attached with continuity at the transition point. N(0, *σ* ^2^) noise was added, where *σ* ^2^ was estimated from the real data (*σ* ^2^ = 0.096) as described below. Curves with more than four pieces were not simulated, since less than 2.5 % of the curves in our data were fit with more than four pieces. PPLs were fit to the simulated data with up to ten pieces.

### Strict pairwise criterion for model selection

After choosing a model using an information criterion, we applied an additional pairwise criterion to remove pieces that may have been overfit. This was necessary because, despite the superior performance of AIC and AICc (see the Results), both exhibited considerable overfitting. To prevent overfitting, each piece was compared to its predecessor by extending both until the end of the curve, removing the five points that the earlier piece fit worst, and using AIC to choose between keeping both pieces or extending the earlier one to both regions. Extending the pieces until the end of the entire curve was an additional mechanism to avoid fitting outliers, since a piece that only fits a short outlier in the middle of the curve would be expected to perform badly on modeling the rest of the curve. The requirement of a lower AIC on the remaining curve, excluding the five worst-fit points from the previous piece, makes splits to two pieces highly unlikely without strong support from the entire curve.

### Estimation of inherent measurement noise

Since our data comprised noisy measurements of “true” skill, even a perfect model of human learning could not fully explain its variance. By itself, the fraction of total variance explained is difficult to interpret, because total variance sums both the variance of the learning process, which a model of learning aims to explain, and the unknown measurement noise unexplainable by a model of learning. To make the results more interpretable, we estimated the inherent measurement noise in our data as follows.

Assuming both an “ideal” curve *x*(*t*) of the learner’s true skill at time *t* and noisy observations *y*(*t*) = *x*(*t*) + *ε*(*t*) with independent and identically distributed (i.i.d.) noise *ε*(*t*) ~ N(0, *σ* ^2^), we estimated the inherent measurement noise by using a first-order approximation to windows of three scores: *x*(*t*) – 2*x*(*t* + 1) + *x*(*t* + 2) ~ 0. In this case, *y*(*t*) – 2*y*(*t* + 1) + *y*(*t* + 2) ~ N(0, 6*σ* ^2^). To validate the assumptions of this estimation procedure, we verified that the null hypothesis of zero mean could not be rejected (likelihood-ratio test, *χ* ^2^(1) < 1.87 and *p > .*17, both for the individual tasks and overall). The standard errors of the noise estimator were small (under 0.0003 for all tasks) due to the large sample size.

The estimated noise was used for the likelihood calculations to be found throughout the article. An alternative method of calculating maximum likelihoods, based on estimating the fit error variance on each curve individually, yielded similar results, so we opted for the more parsimonious approach of estimating a single global noise parameter per task.

### Estimation of variance explained

To estimate the mean squared errors (*MSE*s) for different-size models, we removed one degree of freedom (DF) for every parameter in the model (similar to the commonly used “adjusted *R* ^2^”) and divided the residual sum of squares by the remaining DFs (499 per curve for overall variance; 501 – 5 *k* per curve for a *k*-piece, four-parameter PPL). The variance explained was computed by subtracting the estimated inherent measurement noise from each *MSE* and from the overall variance before dividing.

To validate this procedure, we simulated 100 data sets using four-parameter PPLs with exponentially distributed piece lengths (*λ* = 0.01) and PL parameters chosen in ranges close to values fit to the real data. Gaussian noise was added with variance that was sampled for each simulated data set from an exponential distribution (*λ* = 100). Curves were fit using a single three-parameter exponential function, and the noise estimation procedure described above was applied. The “true variance explained” was recovered with zero bias and *r* > .99 for a wide range of values of “total variance explained” (between .5 and .75), thus validating the above procedure.

## Results

### Simulation study

To choose an appropriate model selection method for this analysis, we compared the widely used AIC, AICc, and BIC (Burnham, 2004) in a simulation study using six metrics (see the Method section). Overall, accuracy was worst for the BIC, due to underfitting multiple-piece curves, whereas the performance of AIC and AICc was superior but indicated overfitting, incorrectly classifying single-piece as multiple-piece curves (Table 2). The predictive performance of AIC and AICc was also superior to that of BIC (Table 3), on all four tasks separately and overall. For the simulated data, AIC, AICc, and BIC all performed identically to a single PL on the single-piece curves, as expected, whereas AIC and AICc performed better than BIC on multipiece curves (Table 3, bottom two rows).

**Table 2:** Evaluation of model selection methods: Evaluation of the simulated data using the six metrics

Metric	Description	AIC	AICc	BIC	spAICc
LL	Negative log-likelihood	1.7009	1.5441	12.4589	
E1	Squared error for best model	1.2425	1.1222	2.0097	1.32567
E2	Squared error for expectation	0.9227	0.8454	1.9876	
ER	Error rate	0.5555	0.5477	0.6530	0.5573
FP	False positives	0.5160	0.4840	0.0000	0.1790
FN	False negatives	0.0510	0.0560	0.4787	0.2227

Lower numbers indicate higher accuracy (see the Method). Akaike weights were not computed for spAICc, so LL and E2 results are not given.

**Table 3:** Evaluation of model selection methods: Percent change in the accumulative prediction error (Wagenmakers et al., 2006; see the Method) relative to a single-power-law fit

Task	AIC	AICc	BIC
2B	−8.03	−8.04	−2.20
1B	−6.49	−6.61	−3.48
EF	−4.03	−3.48	0.05
VF	−3.18	−3.04	−1.16
All	−4.58	−4.48	−1.83
Sim1	0.10	0.07	0.00
SimM	−8.78	−8.45	−4.00

Negative numbers indicate a reduction in error, and zero indicates no change in error. The top five rows are results for real data by task and overall; the bottom two rows are for single-piece simulated data (Sim1) and multiple-piece simulated data (SimM). 2B, two-back memory task; 1B, one-back speed-of-processing task; EF, Eriksen flanker task; VF, verbal fluency task; AIC, Akaike information criterion; AICc, small-sample-corrected AIC; BIC, Bayesian information criterion

Due to the superior performance of AIC and AICc relative to BIC, we chose AICc as the base for model selection, but to prevent overfitting, we applied an additional strict pairwise criterion (see the Method). The final model selection method—*strict pairwise AICc* (spAICc, pronounced “space”)—maintains good overall accuracy (comparable to AICc) with far fewer false positives (Table 2). The fit transition point distances from the nearest actual transition points were strongly centered at 0 (see Fig. 2c below). Exact APE was not computed for spAICc, due to high computational complexity.

### Piecewise power laws

**Fig. 1:**
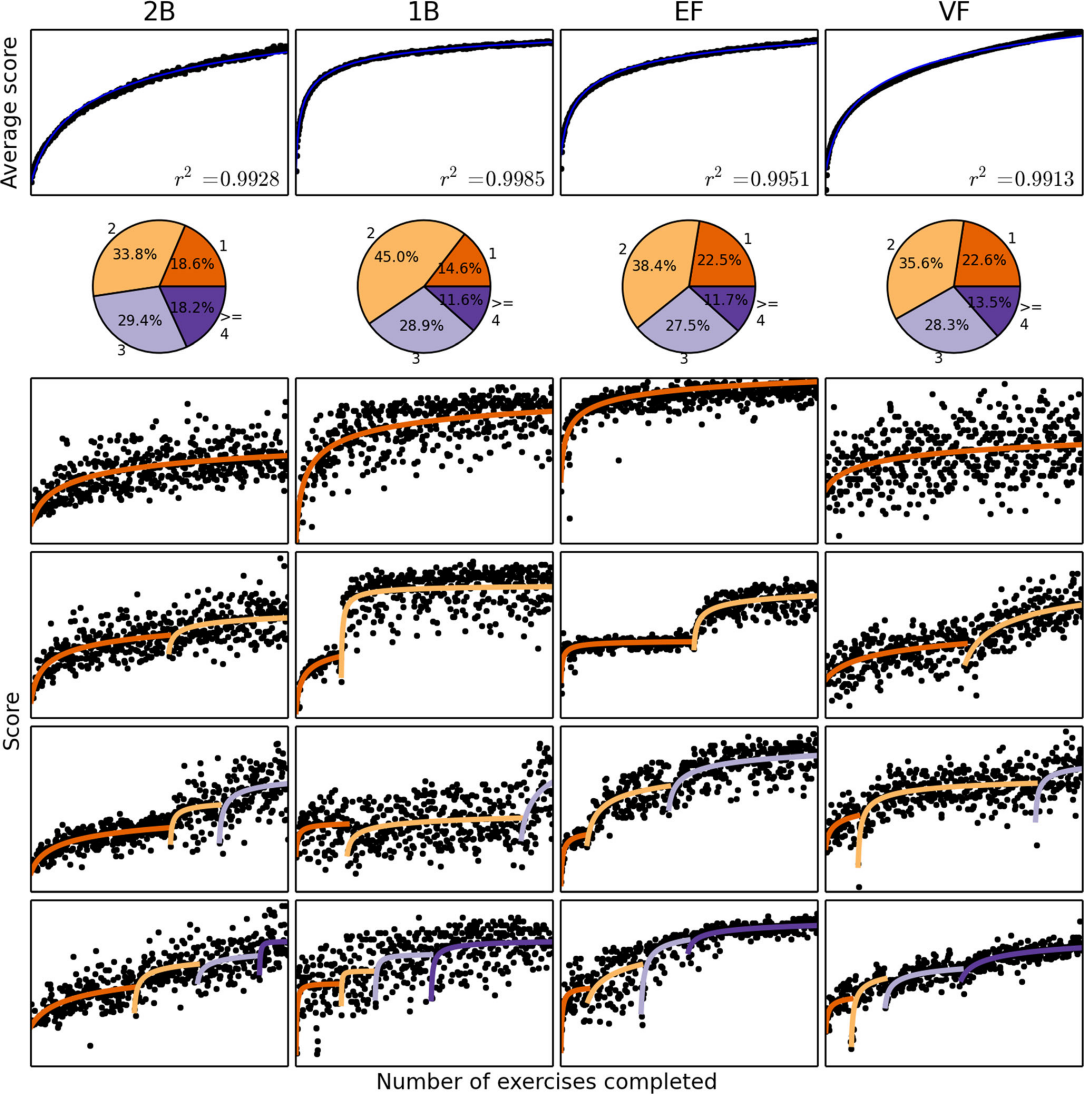
Average and individual learning curves and fits. From left to right: two-back (2B), one-back (1B), flanker (EF), and verbal fluency (VF) tasks. From top to bottom: Learning curve averages (black dots) and fits (colored lines); distributions of numbers of pieces; and example fits with one, two, three, and four pieces (original data, black dots; fitted data, smooth curves, with each piece in its own color). For the learning curve plots, the *x*-axis indicates the number of completed exercises (1–500) and the *y*-axis indicates the score

Consistent with previous reports, the average learning curves in our data were well-fit by a single PL (PL1; *R* ^2^ > .99 for all tasks; see Fig. 1, top). As expected, the best model for the average curves according to all model selection methods contained a single piece.

We fit all individual learning curves using four-parameter PPL with up to ten pieces (Fig. 1, bottom four rows) and PL1. After accounting for the numbers of estimated parameters (see the Method), the PPL fits were superior for all tasks [likelihood-ratio test: *χ* ^2^(1785) = 13,222, *χ* ^2^(8520) = 61,616, *χ* ^2^(9020) = 61,975, and *χ* ^2^(74880) = 367,681, respectively for the four tasks, and *χ* ^2^(94205) = 534,030 overall; *p*s < 10^−7000^ for all tests after Bonferroni correction for choosing one of ten PPL models for every curve]. After correcting for different DFs and subtracting the estimated measurement noise (see the Method), PPL explained 90.74 % of the variance in the data, PL1 explained 86 %, and an autoregressive (AR) model in which each score predicted the next only explained 33.9 %. These results were qualitatively similar for all tasks, with PL1 explaining 78.6 % of the variance remaining after AR, and PPL explaining 34 % of the variance remaining after PL1 (Table 4). Three-parameter PPLs fit almost as well, explaining 90.57 % of the variance, but the small improvement due to the fourth parameter was still significant, even after controlling for increased model complexity (likelihood-ratio test: *χ* ^2^(32731) = 49,514, *p* < 10^−936^). Three-parameter piecewise exponential functions performed similarly to the three-parameter PPLs, but slightly worse, explaining 90.43 % of the variance. Since three-parameter piecewise exponential functions performed worse than three-parameter PPLs on our data, we did not include the four-parameter APEX function (Heathcote et al., 2000), considering the high computational cost of fitting it. For all tasks, two- and three-piece solutions were most common (Fig. 1, second row from top), suggesting that transitions are not frequent, but they affect the curve significantly when they do occur.

**Table 4:** Percentages of noise-subtracted variance (see the Method section) explained by three learning curve models on each of the four tasks and overall

Task	AR	PL1	PPL	ΔPL1	ΔPPL
2B	55.41	88.79	93.75	74.85	44.30
1B	28.89	63.49	71.66	48.66	22.36
EF	38.91	83.49	88.93	72.98	32.92
VF	33.41	91.16	94.97	86.73	43.05
All	33.88	85.97	90.74	78.78	33.99

AR, autoregressive model; PL1, single-power-law model; PPL, piecewise power law model; ΔPL1, fraction of variance remaining from AR explained by PL1; ΔPPL, fraction of variance remaining from PL1 explained by PPL

### Properties of transitions

**Fig. 2:**
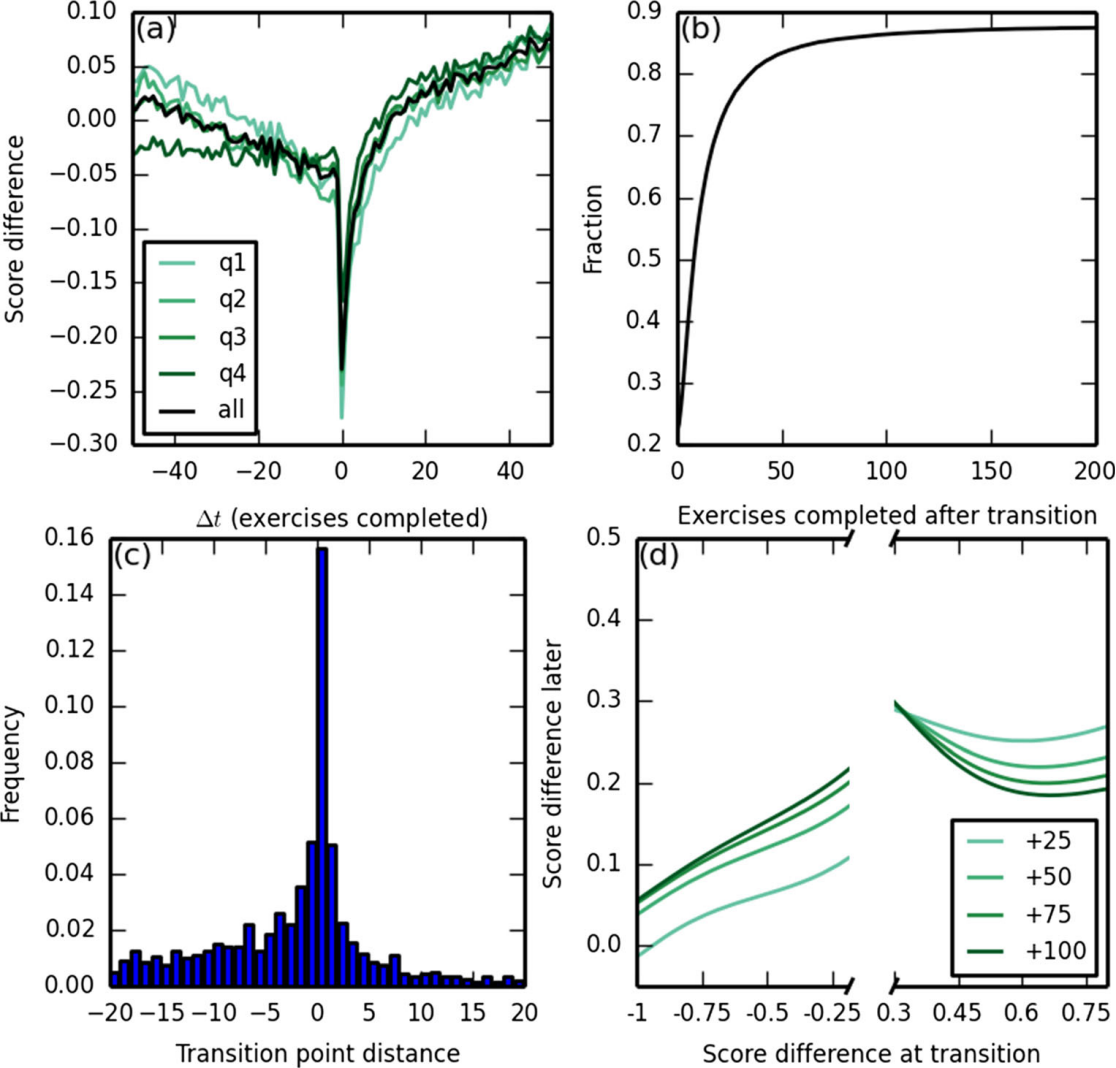
Behavior of power law (PL) pieces near transition points. (a) Average learning curves centered at the transition points, relative to position-matched windows that do not contain transitions, by quartile of total time between completion of the first and last exercises, in a window of radius five exercises around the transition point (first to fourth quartiles in colors; black = overall curve). To control for possible biases of the fitting procedure, the same procedure was also applied to data with the piece order shuffled and the results subtracted (see the text). (b) Distribution (cumulative density function) of the number of exercises for a new piece to surpass the previous piece. (c) Histogram of distances between the fit transition points and actual transition points in the simulated data. (d) Average score differences between the posttransition and pretransition PLs after 25, 50, 75, and 100 exercises, as a function of the immediate score change at the transition point. Score differences greater than zero indicate that the posttransition scores are higher than predicted by the pretransition ones

To characterize the transitions between pieces, we computed the average curves centered at transition points, relative to position-matched windows with no transitions within them. To control for possible biases of the fitting procedure, we performed the same procedure on PPLs fit to control data in which pieces were sampled from PLs fit to the original data, but connected in random order while maintaining continuity at the transition points, and then subtracted the control average curves from the average curves computed on the original data (Fig. 2a). Transitions were characterized across all tasks by a sharp decrease followed by a slightly slower increase to a higher level than before the transition. The posttransition level after the initial performance decrease exceeded the extrapolation of the pretransition curve.

For each transition, we computed the number of exercises it would take the posttransition PL to exceed the pretransition PL, if it ever would (Fig. 2b). Immediately posttransition, performance dropped in 78 % of the transitions, but within 50 exercises, the posttransition PL exceeded the pretransition PL in over 80 % of the transitions.

To investigate the association between the immediate performance decrease and the subsequent increase, we compared the score differences at the transition with the differences 25, 50, 75, and 100 exercises later (Fig. 2d). Moderate correlations were observed (*r*s = .438, .303, .24, and .2 for 25, 50, 75, and 100 exercises later), but the actual slopes were small (Fig. 2d, left; linear fit slopes = −0.2, −0.15, −0.12, and −0.11, for 25, 50, 75, and 100 exercises later). Even when performance did not decrease at all at the transition point, the subsequent increase was observed (Fig. 2d, right).

**Fig. 3:**
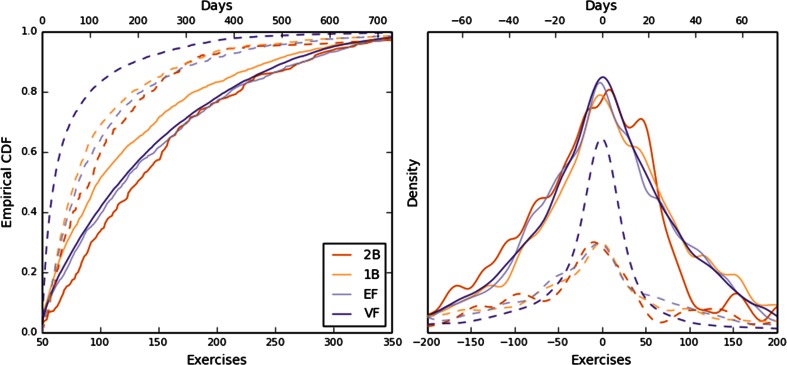
Piece durations. (Left) Distribution (empirical cumulative density function) of the first-piece duration in multipiece curves, measured both by number of completed exercises (solid lines) and days (dashed lines). Colors correspond to the tasks. (Right) Distribution (smoothed probability density function) of duration differences between two adjacent pieces.

In curves containing multiple pieces, the first-piece durations were comparable across tasks when measured by the number of exercises completed and by days, with medians of approximately 125 exercises and 2–3 months (Fig. 3, left). The subsequent piece durations were close to the durations of their predecessors (Fig. 3, right).

**Fig. 4:**
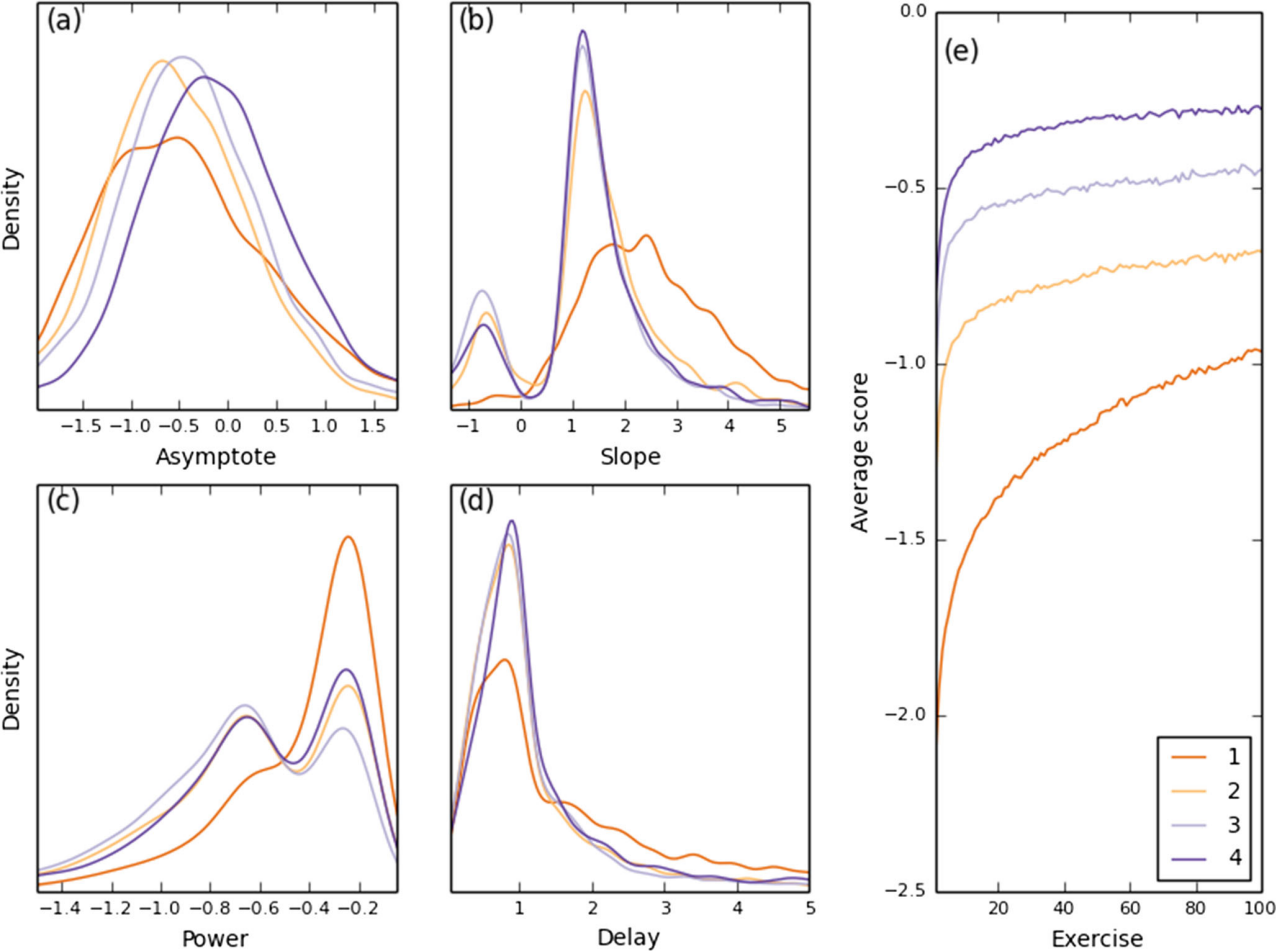
Properties of the first, second, third, and fourth power law (PL) pieces. (a)–(d) Distributions (smoothed probability density function) of PL parameters in the equation *f*(*t*) = *u* – *a*(*t* + *d*)^*c*^. (e) 100-exercise-long piece averages for the first, second, third, and fourth pieces

The global trend of improvement between PL pieces was also evident when averaging the pieces separately by index (Fig. 4e) and in the distribution of PL parameters (Fig. 4a–d). To avoid confounds from the total number of pieces, this analysis was done for curves fit by exactly four pieces. The first-piece parameter distributions differed from those of the parameters fit to later pieces, with a wider range of upper asymptote values, larger scale, and lower curvature. This was also seen in the average of the first piece (Fig. 4e), which rises faster (greater slope) but is less curved (lower power) than the other pieces.

For the pieces after the first, the parameter distributions are similar except for the asymptote, which is slightly shifted to the right for later pieces (Fig. 4a), reflecting the global process of improvement. The delay did not vary much (Fig. 4d), suggesting that it is less important than other parameters, consistent with the good performance of the three-parameter PPL. The slope was mostly around 1, with a small fraction of negative values (Fig. 4b), corresponding to rare cases of decreasing pieces (our model is not constrained to fit only increasing pieces). The power parameter distribution is bimodal, with modes corresponding to low and high curvature (Fig. 4c).

In sum, the sequence of PL pieces is characterized by a monotonic increase in the asymptotes, independent of a commonly observed performance drop at the transition points. This increase reflects a higher-level process of long-term qualitative improvement, separate from local improvements that are well-modeled by the PL function. Besides the increasing trend in the PL upper asymptotes, a decrease in PL slope was observed for later pieces, reflecting slowing of the learning rate. Durations (measured by either time or the number of completed exercises), powers, and delays did not show a strong monotonic trend between PL pieces.

### Associations between demographic variables and learning curve properties

Finally, we investigated the associations between basic demographic properties (age, gender, and education), absolute performance (measured as the mean score over the entire curve), overall improvement (measured as the difference between the ending and start quantiles), and the number of PL transitions. Performance was highly correlated with improvement [*r*(15684) = .8148, *p* < 10^−9000^]. Higher levels of education were associated with better performance and larger improvements, whereas age was negatively associated with both performance and improvement for both genders, and males had slightly better performance (Table 5). After accounting for absolute score and improvement, the number of PL transitions was negatively associated with age for both genders but was not significantly associated with gender or education level (Table 6). The number of transitions was positively associated with improvement and negatively associated with performance (Table 6, rows marked “1”), but this result is difficult to interpret, due to the strong correlation between performance and improvement. We decorrelated them by replacing them with the two normalized principal components “skill” (sum of normalized improvement and normalized performance) and “growth” (normalized improvement minus normalized performance). Since this is an invertible linear transformation, the effects of all other variables remained unchanged. The magnitudes of the effects can be compared since the variables have been normalized. Both were positively associated with the number of transitions, but growth had a stronger association (Table 6, rows marked “2”).

**Table 5:** Demographics and performance: Effects of demographic variables on absolute performance and improvement in a linear regression including all variables

	Performance				Improvement			
Attribute	Est.	*SE*	*t*	*p*	Est.	*SE*	*t*	*p*
Intercept	0.786	0.042	18.53	<10^−15^	0.765	0.013	58.58	<10^−15^
Education: Some college	0.15	0.017	8.846	<10^−15^	0.039	0.005	7.465	<10^−14^
Education: Professional degree	0.205	0.029	7.125	10^−12^	0.049	0.009	5.637	10^−8^
Education: College graduate	0.245	0.017	14.375	<10^−15^	0.067	0.005	12.75	<10^−15^
Education: Grad school	0.307	0.017	17.616	<10^−15^	0.085	0.005	16.03	<10^−15^
Gender: Male	0.093	0.039	2.406	0.016	−0.003	0.012	−0.28	0.777
Age for female	−0.015	0.0004	−35.021	<10^−15^	−0.0049	0.00013	−37.04	<10^−15^
Age for male	−0.017	0.0006	−27.588	<10^−15^	−0.0049	0.00019	−25.67	<10^−15^

*R* ^2^ = .253 for performance, .261 for improvement

**Table 6:** Demographics and performance: Effects of performance, improvement, and demographic variables on the number of transitions, in a linear regression

Attribute	Est.	*SE*	*t*	*p*
1: Performance	−0.51	0.023	−22.433	<10^−15^
1: Improvement	2.14	0.074	28.997	<10^−15^
2: Skill	0.05	0.0052	9.5	<10^−15^
2: Growth	0.382	0.0139	27.49	<10^−15^
Intercept	2.351	0.944	24.883	<10^−15^
Education: Some college	−0.027	0.032	−0.85	.4
Education: Professional degree	−0.075	0.054	−1.385	.166
Education: College grad	−0.053	0.032	−1.647	.1
Education: Grad school	−0.035	0.033	−1.051	.293
Gender: Male	0.088	0.072	1.219	.223
Age for female	−0.009	0.0008	−10.756	<10^−15^
Age for male	−0.012	0.0012	−9.925	<10^−15^

The rows marked “1” and “2” were interchangeable, as we describe in the text. *R* ^2^ = .072

## Discussion

The traditional PL model of practice is highly successful at modeling average learning curves, as is reflected in our data. It does not account, however, for individual-specific changes in learning dynamics due to events or processes that impact the course of improvement. Using a large new data set, we demonstrated that PPL explains individual learning curves significantly better than PL1 does.

To allow us to assert that PPL behavior was a true property in our data, we preprocessed the data to remove outliers and used a conservative fitting and model selection procedure that we validated on simulated data and that is slightly biased to underfitting. Our data strongly supported PPL, but before we may conclude that PPLs are inherent in human learning, alternative explanations need to be ruled out.

Several alternative explanations could account for PPL behavior:

1. Perhaps outliers deviated from a single, smooth learning curve. Since additional PLs could overfit outlier points, we preprocessed the data to remove outliers and used a conservative fitting method to avoid overfitting a small number of outliers.
2. Several participants may have recorded data under the same name, creating a mixture of curves. This is possible, but it is unlikely to explain a robust phenomenon throughout the entire data or the improvements we found in subsequent PL pieces.
3. Discontinuities may reflect a change in performance due to forgetting rather than strategy shifts. Forgetting is a plausible explanation for performance decreases around transitions, but not for improvements in the subsequent PL pieces, which are not explained by outliers or mixtures, either. Forgetting may, however, facilitate switching to a strategy that is better in the long run, and this warrants further investigation.

For the above reasons, globally increasing PPL behavior is likely to be a true characteristic of individual learning curves. This behavior is consistent with strategy-specific PLs that have been previously discussed (Delaney et al., 1998), but we derive this property directly from the data, without assuming known strategies. Our results reveal that strategy shifts are frequently associated with both decreases in short-term performance and improvements in long-term performance. These results are consistent with previously reported effects of between-session delays, including forgetting and improved long-term learning (Rickard, 2007).

The PPL model explained 90.74 % of the learning variance (overall variance minus noise; see the Method), as compared to 85.97 % for PL1, and accounted for over one-third of the variance remaining in PL1. What about the remaining 9.26 %? Quantitative modeling of two additional learning curve characteristics reported in this study could account for some of that remaining variance: the brief discontinuity at the transition point, and the global trend toward increasing PL pieces. PPL fits pieces without learning a distribution to predict them. Moreover, other parametric forms may better apply to the individual curve pieces, such as four-parameter APEX functions (Heathcote et al., 2000), which we did not include due to high computational cost and the lower performance of three-parameter exponential functions, relative to three-parameter PPLs. Since the performance differences between different parametric forms in our experiment were small relative to the differences between PPL and PL1, our focus in this work was on investigating the piecewise nature of individual learning curves rather than specific functional forms to fit each piece. Hierarchical models could account for even more variance by specifying piece parameter distributions as being dependent on higher-level parameters describing the relationships between pieces in a curve. It is likely that additional processes can account for more missing variance, and we hope that future studies will reveal these processes.

A full analysis of the influence of spacing in time on performance and learning is outside the scope of the present study. However, since the average transition curves were similar for all quartiles of the total duration in a window of five exercises before and after the transition, spacing was not a major factor in the transition behavior we report here.

The PPL of practice extends learning theory and suggests specific directions for future investigation, as we outline below. Practically, considering learning progress both locally, within smooth PL trajectories, and globally, within sequences of discrete improvements, could improve skill acquisition through data-driven planning of training. Future discoveries about transitions could further accelerate learning by triggering transitions at specific times. For example, if theory or data suggest that certain advanced strategies depend on the preexisting mastery of simpler strategies, learners could track progress on each PL and transition at appropriate points, thus avoiding both superfluous practice on earlier strategies and premature attempts to acquire later ones. The acquisition of expert performance follows similar principles (Ericsson, Krampe, & Tesch-Römer, 1993), further supporting our results.

In this study, we have shown that individual human learning curves in natural learning settings follow a PPL sequence in which subsequent PLs tend to surpass earlier ones, usually after a temporary drop in performance. Many open questions remain, including: What are the factors causing transitions between PLs? What is the relationship between the temporal spacing of training sessions and the learning curve? Why are transitions usually accompanied by a temporary drop in performance? And what regularities in global PL sequences exist within and across tasks? We hope that future work will answer these questions and further improve our understanding of individual human learning curves.

## Appendix

### Python code for our PPL fitting algorithm

      The Python code a[j:k] means “positions *j*, *j* + 1, . . . , *k* – 1” in the vector **a**. The code a[j,:] means “the entire row indexed by *j*” in matrix **a**. The expression range(j) means 0, 1, . . . , *j* – 1.

def AdvanceByOne(u, a, c, d):

return (u, a, c, d+1)

def ComputeWindowErrorMatrix(curve):

l_curve = len(curve)

M = zeros([l_curve-min_window+1, l_curve-min_window+1])

for i in range(l_curve-min_window):

if i > 0:

warm_start_parameters = AdvanceByOne(previous_parameters)

fit = FitPowerLaw(curve[i:i+min_window], warm_start=warm_start_parameters)

else:

fit = FitPowerLaw(curve[i:i+min_window])

previous_parameters = fit.parameters

M[i,0] = fit.error

warm_start_parameters = previous_parameters

for j in range(l_curve-i-min_window):

fit = FitPowerLaw(curve[i:i+j+min_window+1], warm_start=warm_start_parameters)

warp_start_parameters = fit.parameters

M[i,j+1] = fit.error

return M

def FitPiecewisePowerLaw(curve, min_window=50, max_pieces=10):

l_curve = len(curve)

M = ComputeWindowErrorMatrix(curve, min_window)

P = zeros([max_pieces, l_curve+1]) + infinity

B = zeros([max_pieces, l_curve+1])

P[0,:] = M[0,:]

for i in range(1, max_pieces):

for j in range(1, n+1):

vals = P[i-1,0:j] + M[0:j,j]

argm = argmin(vals)

P[i,j] = vals[argm]

B[i,j] = argm

best_paths = [None] * max_pieces

for pl_i in range(max_pieces):

best_paths[pl_i] = [l_curve]

j = pl_i

while j > 0:

best_paths[pl_i].append(B[j,best_paths[pl_i][−1]])

j -= 1

best_paths[pl_i].append(0)

best_paths[pl_i].reverse()

return P, B, best_paths
